# Exploring the experiences of the older adults who are brought to live in shelter homes in Karachi, Pakistan: a qualitative study

**DOI:** 10.1186/s12877-019-1376-8

**Published:** 2020-01-06

**Authors:** Laila Akber Cassum, Keith Cash, Waris Qidwai, Samina Vertejee

**Affiliations:** 1 Aga Khan University School of Nursing and Midwifery, P.O.Box 3500, Stadium Road Karachi, Karachi City, Pakistan; 2 Faculty of Public Health, Kuwait University, Hawally, Kuwait; 3 Department of Family Medicine, Karachi City, Pakistan

**Keywords:** Older adults, Shelter homes, Experiences, Pakistan, Challenges, Institutionalization, Mini, Mental State Examination, Qualitative study

## Abstract

**Background:**

The traditional joint family system in a culturally diverse Pakistani society shows great respect and care for older population by the families and their generations. However, in the recent years the phenomenon of population ageing in Pakistan is rapidly increasing due to demographic shift influencing life expectancy, along with changes in socio-cultural values. This transition has resulted in institutionalization of the elderly as an emerging shelter alternative. The aim of this study was to explore the experiences of the elderly people and to identify the reason which compelled them to reside in these shelter homes.

**Method:**

A qualitative methodology, with a descriptive exploratory design, was adopted for the study. A purposive sample of 14 elderly males and females were selected, from two different shelter homes in Karachi, Pakistan. Semi-structured interviews were audio recorded and transcribed. Content analysis was done to extract the themes and comprehend the data.

**Results:**

Content analysis revealed five major themes: the circumstances of leaving home, experiences, and challenges to wellbeing before entering the care facility, coping with challenges, and decision to live in a shelter home. The analysis discovered that the elderly were experiencing lack of physical, psychological, emotional, and financial support from their family and children. It also indicated that migration of children for better career and employment opportunities, entrance of women into the workforce, and insensitive behaviour of children, left the senior citizens neglected and helpless. The findings also uncovered the challenges of unemployment and family disputes that the elderly had to face made them dependent, distressed, helpless, and lonely resulting in both their apparently willing and forceful decision to reside in shelter homes.

**Conclusion:**

The findings point to need for further investigation of the identified areas in this study through qualitative and quantitative researches. There is a dire need for increasing public awareness through the social, electronic, and print media, and providing capacity building training to HCPs for the care of the elderly. The lobbying group can act as a catalyst in persuading the government officials for the execution of a policy on retirement, day care and subsidized provision of health services for the betterment of the elderly.

## Background

The ageing population is a significant and universal global trend [1, 2]. From being a feature of developed economies, it is now seen in developing countries that are moving towards a more global economy. One result of this move has been a change in the socially derived roles normally attached to older adults in these countries. In a traditional society, with low population mobility, entrenched gender roles, and an extended family structure, the elderly are given a social status; that same is often not the case in the more developed global economies.

Globally, the elderly population is growing at a startling rate. A WHO report on global health and ageing estimated that, “In 2010, 524 million people were aged 65 or older, 8% of world population. By 2050, this number is nearly expected to triple to about 1.5 billion, representing 16% of the world’s population [3]. Geographically, the rate at which the population is ageing is much quicker in the developing countries [4], with Asia being the continent with a large number of people in the age band of 60 years and above [5].

Life expectancy in Pakistan has increased by approximately thirty years in the last 50 years, and it will reach 72 years by 2023 [6]. Pakistan, being the world’s sixth most populated state, currently has more than 8 million older adults and this number is expected to reach 27 million by 2050 [7]. In addition to the demographic shift, economic recession, inadequate saving levels, lack of health insurance coverage, and a weak pension system are affecting the lives of this vulnerable group [8].

The joint family system has been the prevalent family structure in Pakistan, in which people respect and value the older people [9, 10]. As per this system, older adults have traditionally been cared for at home by their families, where they expect love, respect, and admiration from their children. Similar to many of the South Asian countries, Pakistan has a diverse society that is multicultural, multi-ethnic, and multilingual [11], where culture, traditions, values, and family bonding, until recently, were given high importance. However, in many South Eastern Asian countries, the elderly in Pakistan, are confronted with a decline in the traditional extended family system and the emergence of nuclear families [12–15].

The escalating trend of urbanisation and industrialisation is resulting in the migration of younger individuals for better employment and professional opportunities, the rise of career-oriented families, the entrance of women in the workforce, delayed marriages, and an increasing number of childless marriages [14, 16, 17]. All these factors have resulted in the erosion of traditional caring and support provided by the extended family members.

Another important issue in Pakistan is the weak and fragile system of pension distribution after retirement. The retirement age in Pakistan is 60 years for males and 55 years for females [8]. Only a small proportion of older adults are financially independent after retirement. The main reason for this is the lack of government funds because of the weak tax base [8, 14]. This has condensed the centrality of the older persons in the family, which has resulted in lack of physical, psychological, emotional, and social support provided by the family members [9, 10, 16]. Due to these changes an increasing number of older people will be obliged to either spend the last years of their lives in increasing isolation or in shelter care facilities.

According to the social welfare department of Sindh, six organizations are giving shelter protection to more than 150 older people [18]. The actual number is much more than the reported figures. In Karachi, these homes are managed by private NGO’s or faith-based organisations. For example, the Catholic Church manages several shelter homes in Karachi, where the residents pay a minimal fee, and some are managed by other faith-based communities, functioning with the help of volunteers and donation support [14]. The Edhi Foundation is an example of a service provided by an NGO where abandoned older adults are housed [13]. Approximately, 417 residents at the Edhi old home are reported to be benefitting from this organisation, although the actual number is under-reported [19].

The notion of shelter homes is still in the initial stages in many of the South Asian countries and it appears that there is no previous study on older persons, who have to relocate in these shelter homes, in Pakistan. Hence, there is an urgent need to research the real experiences of the elderly, brought to live in these shelter homes so that quality of life of this age group can be enriched.

### Study purpose

The purpose of this study was to explore the experiences of older adults who are brought to live in shelter homes in Karachi, Pakistan, and to identify the reasons for their admission to these institutions. The specific questions were:

1. What circumstances and experiences contribute to older Pakistani people seeking residence in shelter homes?
2. What are the issues and challenges faced by the older persons in their lives that led to their relocation in these homes?
3. How do these older persons cope with the move to the institutions?

## Methods

### Study design and setting

A qualitative methodology, with a descriptive exploratory design, was chosen for the present study, because of its capacity to explore the narrative accounts of the life experiences of the older adults who come to live in these shelter homes. A qualitative approach with descriptive exploratory design explores the life experiences from people’s perspective to get a new insight and understanding of the phenomenon [20–23]. This approach was considered useful as there is little pre-existing data on the subject in the Pakistani context.

Two care facilities located in Karachi, Pakistan, were selected for this study out of the six available. These shelter homes were selected to provide a broad range of interviewee experiences. The information about the two shelter homes given below, is not a comparison but the facts have been stated as provided by the respective authorities.

### Shelter home A

This care facility operates under the leadership of a faith-based community. This home provides accommodation for approximately 70 older adults of both the genders. They are admitted on the criteria that, 1) there is no family member or another caretaker available to take care of the person because their children live permanently in different countries, 2) because of family conflicts and 3) psychological issues. The care facility charges approximately Rs. 23,000 ($164) per resident per month; however, if the person cannot afford this then the Social Welfare Board (SWB), and the donors of this particular religious community provide the funding based on assessment by SWB. The facility is neither registered by the government nor is it supported by it. (personal communication, March 19, 2017).

### Shelter home B

This home is also operated by a faith-based community and is not registered with the government. It provides services to approximately 40 older adults of both genders based on the criteria of 1) rejection by the family and 2) a letter of recommendation by the local leader of this particular religious community. It operates on a charity basis and has no government support. The home charges Rs. 2000 per month for the accommodation and other expenses (personal communication, March 19, 2017).

#### Sampling and sample size

The study used the maximum variation purposive sampling technique, selecting cases that came from diverse backgrounds and circumstances [24, 25]. Based on the inclusion criteria, 14 participants were recruited from the selected nursing homes. The criteria were: aged 65 years and above, with the ability to recall and describe experiences, speaking English or Urdu, currently living in a care facility, and willing to participate in the study. The older adults, with severe cognitive impairment (scoring 0 to 10 on the MMSE) [26], and severe hearing difficulties were excluded from the study.

#### Demographic profile and MMSE scores of study participants

The demographic profile of the study participants is illustrated in Table 1. The study sample comprised of 14 participants, including eight males and six females. Their ages ranged from 65 to 83 years, with the median age of 74 years. The majority of the participants [12] were married, and two were unmarried. Out of the 12 married participants, there were eight participants whose spouses were not alive, two had been separated and two participants’ spouses were alive. 78.6% of the participants had lived in a nuclear family, while 21.4% came from a joint family setup. The participants’ educational profile showed that nine participants had attained primary education, while four had studied up to matriculation (Grade X), and one received education from the Cambridge system.

**Table 1:** Demographic Characteristics of Study Participants

Variables	*n* [14]	Percentage (%)
Age		
65–75 years	8	57.1
76–85 years	6	42.9
Gender		
Male	8	57.1
Female	6	42.9
Marital Status		
Unmarried	2	14.3
Married	12	85.7
Spouse Status		
Alive	2	14.3
Dead	8	57.1
Separated	2	14.3
Family Structure		
Joint Family	3	21.4
Nuclear Family	11	78.6
Number of Children		
0	3	21.4
1	3	21.4
2	2	14.3
3	2	14.3
4	3	21.4
7	1	7.1
Number of Boys		
0	6	42.9
1	3	21.4
2	2	14.3
3	3	21.4
Number of Girls		
0	5	35.7
1	5	35.7
2	2	14.3
3	1	7.1
4	1	7.1
Education Profile		
Primary	9	64.3
Matriculation	4	28.6
Cambridge	1	7.1

The MMSE scores are illustrated in Table 2, ten participants out of 14 were within the normal cognitive score range of 27–30, while three participants’ were under the category of mild cognitive impairment, with a score range of 21–26, and one participant was classified under moderate cognitive impairment with a score range of 11–20 on the MMSE instrument. The participants who fell under the category of mild cognitive impairment were having issues with thinking, finding words, concentrating, and reasoning, whereas the one who was identified with moderate impairment was having problems in the domains of memory, attention, judgment, and reasoning.

**Table 2:** *Mini Mental State Examination Scores*

Scores out of 30		
20	1	7.1
24	1	7.1
26	2	14.3
27	3	21.4
28	1	7.1
29	3	21.4
30	3	21.4

### Ethical considerations

Approval for the study was obtained from the Aga Khan University Ethical Review Committee was obtained. Access to the care facilities was granted by the stake holders of both the care facilities. The purpose, objectives of the study, and the methods of data collection were verbally explained to the participants who agreed to participate in the study. The anonymity and the confidentiality of the shelter homes and the participants were safeguarded by assigning pseudonyms. The participants were informed about their rights to refuse and/or withdraw from the study at any time without any justification.

### Data collection

Data was primarily collected through formal one-to- one in-depth interview using a semi-structured interview guide. During the process, the researcher utilized different strategies like field notes, observations, and reflective journals to assure the validity of what is heard and observed during the encounter with the participants however, these were not the part of triangulation of analysis. In-depth interviews captured complete and detailed information about individuals’ thoughts, feelings, and genuine lived experiences [20, 21, 27].

Field notes were taken before, during, and after the interview session to record the participants’ nonverbal clues, mood and attitude, gestures, and facial expressions. Field notes are primarily the observations and assumptions captured by the investigator of what is heard and observed during the encounter with the participants [27]. These notations played a significant role during the data analysis process, as they served as a valid justification for interpreting data and highlighting crucial emerging notions [27]. Reflective journals were maintained to identify researcher’s personal impressions and interpretations and bracketing of preconceived beliefs and opinions to confront the data in pure form [28]. The field notes and reflective journals were written the same day after the interview and were maintained in a separate diary.

#### Interviews

Formal one-to-one in-depth interviews were conducted, using a semi-structured interview guide. The guide was translated into Urdu. Each interview lasted for approximately 30–60 min, and was audio recorded. A short debriefing meeting was held with the individual informants to evaluate the process of the interview, and to highlight the areas stated by the participants in their comments could be integrated into the following interviews. The semi-structured interview guide and the MMSE instrument were piloted on two participants, each belonging to a different care facility. The actual in - depth interviews were started with broad open ended questions like, “What were the particular circumstances you experienced that brought you to live in the shelter home?” and “How did you cope up the separation from your families?” These were supplemented with further possible probing and leading questions to have a guided conversation with the residents. The interviews were transcribed into Urdu and then translated it into English.

#### Data analysis

Qualitative content analysis is the strategy of choice in qualitative descriptive studies, where data analysis is performed synchronously with data collection [23, 24, 27, 29, 30]. Moreover, qualitative data analysis is an iterative process of submerging oneself in the data and getting an in-depth understanding of it [22, 29].

Data Analysis was done concurrently with the data collection. Rigorous content analysis of data was done by following the guidelines given by Miles and Huberman [29]. The process involved reading and re – reading of transcribed data word by word and sentence by sentence, to get the maximum insight before breaking it into parts. Firstly, the texts were systematically collated for each broad question for example, the responses for question “reason for leaving own home and living in a shelter home” were arranged in a document with three columns. The left column was titled as “codes”, the middle column as “informants’ verbatim”, and last one as “thoughts and comments while the reading text”. Then, the collected data under that particular question was analysed to develop codes. The *in – vivo codes* were identified by highlighting them with different colour marker from the text, and assigned a tag, word or phrase to them that gave meaning to the segment. The codes were written in the side-lines of the transcript to examine for redundancy and similarity. Once the coding of all the transcribed interview was completed, categories and sub categories were created through clustering codes with similar ideas. Finally, themes were extracted that articulated more with the concepts expressed in the text. The entire team was consulted by the researcher to ensure the interpretation were valid, trustworthy and contextualized in participant’s broader perspective. The researcher also kept discussing with the supervisor until the themes adequately reflected participant’s viewpoint. Saturation was attained when no new data provided additional codes, categories and themes. Final inferences were not drawn until all the data had been collected, recorded and analysed with genuineness.

## Results

**Fig. 1:**
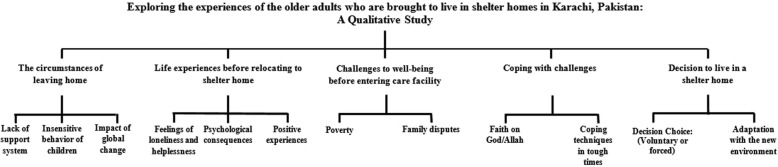
Flow chart of themes and categories

The flow chart of themes and categories is illustrated in Fig. 1. The content analysis revealed five main themes: the circumstances of leaving home, life experiences before relocating to shelter homes, challenges to wellbeing before entering the care facility, coping with challenges, and decision to live in a shelter home.

### Theme 1: The circumstances of leaving home

This theme emerged when the participants were inquired about the reasons and circumstances for their relocation in old age homes. Three main categories emerged under this theme: lack of support system, insensitive behaviour of children, and the impact of global change.

#### Category 1: Lack of support system

A wide majority of the participants were desperate to share their experiences of not being provided with the physical, emotional, and financial support from their children, as they were growing old and were dependent on them for food, shelter and clothing and activities of daily living. The elderlies also expressed that, despite fulfilling the responsibilities of educating, bringing them up until they were adults and settling them down, the children did not “pay back” in terms of meeting their parents’ expectations. The children were unwilling to care and deprived their parents from basic necessities of life. A participant cried while narrating his feelings:

She never gave me food…. At times I survived on an empty stomach for days… I stood up and told them after three days of staying on an empty stomach. They were eating chicken biryani (Eastern dish made of chicken and rice with spices). I told them to give me if there is some left after all have been fed. My granddaughter crawled towards me and offered me a piece of bread. Then my daughter-in-law said that, they will give me some if anything is left ….. Otherwise she told me to go to sleep (NB-14).

A depressed father who was very disappointed with his children reluctance to support him shared, “At this time, I do not have any family to support me and no roof to live under. I don’t know where I would have gone; whether I would have spent my life on a railway station or on a foot path. I didn’t know…” (MC-6).

The parents in South Asian culture especially focus on educating their male children, so that they can depend on them for financial support in difficult times and old age. They also expect to willingly fulfil their obligations and assist them financially when there is no source of earning. An upset father said, “I stayed here and took care of the family. I sent him abroad…So that he can support and assist us financially in times of need, when we get old. I have never requested him to support me and send the money…….He knows everything and is well aware about my condition” (JM-2). The participants also revealed challenges like arranging for finance when their spouse was sick and urgently required money. They were forced to live in misery and pain, and had to utilize their savings and assets to meet their financial needs. The participant explained, “My wife’s condition got so serious that I took her to a hospital where she was kept for a month…. I even had to sell my house for her operation” (AA – 1).

#### Category 2: Insensitive behavior of children

This category sheds light on the children’s insensitive behaviour towards their parents. The participants displayed nonverbal clues like taking long pauses, and getting into frequent crying spells when they were inquired about the behaviour of their immediate family members at home. Two of the participants requested to pause the recorder, so that they could cry out loud before continuing with the interview.

There was lack of acceptance in the role of father, mother, father-in-law, or mother-in-law by their children and their spouses. In eastern culture, these roles are the symbol of respect and deserve high esteem by the children and their families. They encountered profound resentment by the family, and were criticized on petty issues. The participant reported rude and harsh comment of his daughter in law, “When I told her that I am leaving, she said, “Who has stopped you from going… It was clear that she did not love me and did not want to keep me. So I left my home” (MR-12). The magnitude of resentment of a daughter for her father can be seen in this excerpt. “She abuses me, she calls me shameless [*Woh gali galooch karti hai, baigairat bolti hai*]. I am her real father and not a step one. She should not be so sharp and sarcastic while speaking” (MC - 6). The parents are the ones who bring up the children, teach moral and ethical values, and once they are in their ageing phase, the children neglect them. The children and their families considered the elderly members as burden and it was tough for them to tolerate their agonizing comments and severely insensitive behaviour. The participant recalled in a grief stricken state:

My daughter- in- law behaved badly with me. She used to say, who will take care of her if she falls sick? [*yeh beemar par jayegi tau is ka kaun karay ga*], who will give her a bedpan? [*bedpan kaun deyga*]. She taunted and asked me to die and return to God and misbehaved with me all the time [*Jehan teri behan chali gai hai tu bhi wahan chali ja*] (MR-6).

This category also reflected that the male children of the family were often torn between filial obligations and with their new family commitments and responsibilities after marriage. In order to meet the expectations of parents and spouse, the son of the family gets under spousal pressure and consequently, misbehaves with the elderly parents, who raised them up. The participant recounted, “He has bent himself in front of his wife’s command. [*Uss nay apnay aap ko jhuka diya hai*]. He feels embarrassed [*Woh sharminda hai*]. My elder son now regrets a lot [*Mera bara beta bohat pachtata hai*], that despite having a son his father is living in an old home” (JS-11).

#### Category 3: Impact of global change

This category presents the findings related to how globalization has transformed and influenced the younger generations. The impact was evident as the children migrated to the western countries for pursuing higher education and better earning prospects, leaving the elderly parents without support. An informant explicated,

He lived in Pakistan for four months. He tried to search for jobs, so that if he could find an appropriate job he could stay here and take care of his mother as well. He did not get the required one. But there, he is earning more. (RS-13).

The findings also underlined a significant impact of globalization in eastern families, which was seen as a shift in caregiving role of a women, from a traditional house hold role to an empowered and self – reliant role. Work life left no time for women to perform the usual and expected care for the elderly at home.

### Theme 2: Life experiences before relocating to shelter home

This theme emerged when the participants were asked to describe their life experiences which led them to relocate into shelter homes. The negative experiences emerged significantly however, few positive experiences were also shared by the participants. The rich life experiences which emerged were feelings of loneliness and helplessness, psychological consequences and some positive experiences.

#### Category 1: Feelings of loneliness and helplessness

In this category, majority of the participants reported their feelings of isolation, which were resulting from terminal illnesses of spouse, death of spouse or children, and marriage of their daughters. They needed someone to talk and share their concerns and stressors. The participant explained in these words, “I need people around me. Like, when I and my husband lived together, he was there with me. After his death, I need someone with me; I don’t like to live alone” (FR-5).

A few of the informants reported feelings of isolation at their parting with their daughters, at the time of their marriage. Marriage is a cultural custom in Pakistan, where a girl becomes a part of husband’s family after the wedding. Besides, a few of the participant’s reported extreme loneliness on the death of their children. An elderly mother, who had lost her two sons, reported sadly, “My first boy died in 1999 and my second boy died in 2009, exactly after ten years. The deaths of my children left me all alone in this world” (MR-7).

This category also revealed that, cultural constraint, fear of society’s criticism of residing with their married daughters and merciless behaviour of children were the reportable reasons of the elderly participants in a helpless state. A helpless father stated his views by saying,

She will take me, but I do not want to go. The reason is that she is my daughter and in our society it is not considered appropriate if a father lives in his daughter’s house after her marriage [*humaray muasharay main acha nahi samjha jata kay baap beti kay ghar ja kaar rahay*]. I do not want to be a burden on them [*Main unpar jaa kar bojh banoo, yeh nahi chata*], or may be in future I do not want them to say that I am a burden for them. I don’t want her husband to taunt her because of me [*Main nahi chata kay meri beti ko iska khavind taanay* maaray]. (AK-10).

The informants’ conveyed that their lives were made miserable by their children and their spouses, leaving them in a more vulnerable state. A participant narrated his emotions, as he did not want to leave the house because of his intense attachment with his grandchildren and his own physical disability. He said, “I became homeless after the death of my son, there was no place to live….[ *Meray betay ki death kay baad main baighar ho gaya*]. My daughter-in-law told me to make some arrangement for myself at some place. We cannot keep you…That day I cried a lot [*Us waqt meri bahu nay kharay ho kar kaha kay papa, aap apna kahin aur bandobast karlo hum aap ko nahi rakh sakti…Uss din main bohat roya*]” (NB-14).

Few participants reported their helplessness, as they were physically and financially dependent on their children, and were also willing to work and earn in old age but could not find a job. A helpless participant said, “At this age, people don’t employ us, we don’t find jobs. I don’t have money. I am unable to find any employment at this age. I was trying to hunt for work but couldn’t find a job….” (MC-6).

#### Category 2: Psychological consequences

In this category, majority of the participants reported various psychological concerns such as development of fear, anxiety and depression on being lonely and helpless. One of them voiced her anxiety by gesturing towards her chest and heart. She expressed, “After the death of my husband, I get too anxious and worried because of loneliness [*Akaylay pan say bohat ghabraahat hoti* thi]” (FR-5). Many of the elderly articulated about depression after the loss of loved ones as there was no one to care and hear from them. An elderly lady reflected, “I was very upset and went into depression after my daughter went. I felt as if I lost something precious [*Meri beti chali gai tau mujhay bhari para, depression ho gaya. Mujhay lagta tha kay meri koi qeemti cheez kho gai ho*]” (RS-13).

#### Category 3: Positive experiences

This study also unveiled positive feelings shared by few elderly informants about their family and children. The children were concerned for their parents but were helpless and had to leave their parents alone with a heavy heart. A few participants stated their close bonding and caring attitude with all the family members. One of the participant reported,

My daughter-in-law didn’t want me to leave but I preferred to stay separately. You see, now she is a widow. She is young and she may be wanting to have some friends; if I am there she would hesitate to call any friends so that’s why I said: No, I think I will go and stay in a shelter home but we will meet each other frequently (MR-7).

### Theme 3: Challenges to well-being before entering care facility

This theme surfaced, when the informants were asked about the reasons and circumstances which brought them to live in old age homes. The significant challenges encountered by the elderly people have been categorized into poverty and family disputes.

#### Category 1: Poverty

Majority of the participants belonged to a low socio economic group and had faced extreme financial challenges in their lives. Because of unemployment and illiteracy they were unable to educate themselves and their children and find reasonably paying jobs. Additionally, because of lack of money, it was challenging for them to manage domestic expenses, health, education, and other costs of living, and were in a complete helpless state. A participant reflected, “At this age, people don’t employ us. We don’t find jobs. I was trying to hunt for work but could not find work to manage my household living” (MC-6).

#### Category 2: Family disputes

Family dispute was another challenge that emerged from this study. The conflicts usually stemmed from issues of children’s marriage, parenting, unwilling to keep elderly parents at home and caring for them, and non-provision of food and shelter, etc. The elderlies considered these conflicts as the reason of distancing of family members and loss of family bonding and harmony between elderly’s and family. The participant’s disclosed feelings of frustration which were arising because of family disputes and vice versa. The participants were annoyed with children and family member’s behaviour, and had no choice but to live in an old age home. An irritated elderly father narrated his feelings for his daughter-in-law. He said, “My daughter in law challenged me that she will not let me live here in this house. She said that she will destroy my marital life or her own” (JS-11).

### Theme 4: Coping with challenges

This theme emerged when the participants were asked to describe how they managed with the challenges and stressful life situations. Almost every elderly participant reported praying in tough times and considered it as a source of spiritual support and relaxation. Apart from the regular offering of prayers, recitations of verses from holy books, like “The Quran” and “The Bible”, “Tasbeehats”(reciting the rosary), and “reading of religious books” provided them inner peace and serenity and assisted them during the times of stress. The richness found in their genuine expression has been categorized into faith in God/Allah and coping techniques in tough times.

#### Category1: Faith on God/Allah

Majority of the participants had an immense faith in religion, and this conviction was a major source of strength when they were battling with their life stressors. A participant expressed his blind faith on religion, “I just know that Allah is the one who assists you every second and He will respond too because He is always with you [*Bas main yeh jaanta hoon woh har paal saath dainay wala hai*]” (QM-3). Another participant expressed her feelings of satisfaction in the following words, “I opened up the BIBLE and it was written that your brothers and sisters will leave you but I will not. I got immense satisfaction after this and prayed to God and thanked Him” (MR-12).

#### Category 2: Coping techniques in tough times

This category revealed various coping strategies, which a majority of the participants turned to during the difficult times of their lives. What topped the list amongst the strategies was prayers; others included crying, and positive thinking. These strategies gave them strength, inner peace and tranquillity. Prayers were recited as a religious norm and to stay away from worldly stressors. A participant remarked, “Whenever I get afraid, I recite my prayers [*tasbeehs*]” (DL-4). Many of the participants narrated their close connection with God/Allah particularly in tough times of their lives. Another remarked, “I remain connected with God and holy sayings. When I am sitting, even at that time I recite God name internally” (AK-10). Some of them also voiced about the reading of religious books and holy verses during stressful times and he stated, “When I am in difficulties and problems, I read religious books [*mazhabi kitabain*]. It guides you to a solution of the problem” (QM-3). Additionally, positive thinking and sole belief on God/Allah supported them in handling their life stressors however, few of the residents considered crying as a technique to cope, and they felt strengthened after giving a vent to their feelings. One of the elderly stated in a hopeful voice, “I have never been down. I have always been optimistic and always look at the bright side of things. I never thought that something bad will happen tomorrow because God is there and He is looking after me…” (MR-7).

### Theme 5: Decision to live in a shelter home

The final theme emerged, when the participants were asked to express their feelings when they made the decision to move to a shelter home, whether voluntarily or against their wishes. They were also asked about how they felt about living and adjusting in a new environment, away from their families and their loved ones. Their genuine experiences have been categorized into: decision choice: (voluntary or forced) and adaptation with new environment.

#### Category 1: Decision choice: (voluntary or forced)

Majority of the participants shared that, it was their choice to leave their homes and none of the family members forced them to opt for a sheltered home. Nevertheless, there was no other choice left for them when their children did not want to keep them and take care for them. Hence, they felt it was better to leave their homes and dwell in substitute shelters. These participants had experienced a lot of loneliness, neglect, and rejection, and what they construed as insensitive behaviour of their children. As one of the elderly described, “Nobody forced me. I came on my own. I was left like an unwanted person. I don’t want to live in that house” (JS-11). Another expressed freedom from long standing family issues. She narrated, “I have worked like a donkey for my family …. I was fed up and was feeling miserable. How much could I do? I am happy that I came here” (JD-8). Additionally, few of the participants expressed that they were forced to leave their homes by their immediate family members. The father painfully expressed these words, “Many times, my daughter bluntly told me to get out of the house along with my belongings [*Kitni baar kaha niklo ghar say, iska samaan nikalo baahir*]” (MC-6).

#### Category 2: Adaptation with the new environment

This category presents adjustment concerns when relocated into shelter homes. These elderly people were emotionally attached with their home where they lived their entire life. Though, the participants had left their homes, the memories of their home and family were still fresh in their minds and they got distressed and emotional when they reminisced about their past experiences. One of the participants sobbed and verbalized, “I left that house with a heavy heart. I have lived my life there. [sad] [crying]” (FR-5). Another participant disclosed mixed feelings while leaving her home and stated,

I was feeling very sad. I had lived in that house for thirty three years but at the same time, while I was there my daughter got married my son got married my other son got married and then I lost my two boys all in the same house. So there were good memories and sad memories. (MR-7).

These elderly participants also encountered difficulty in adjusting with the old age home environment. The new surroundings, changed physical location, different daily routine and pattern affected their comfort and adjustment level. Almost all the informants communicated difficulty in accepting the new environment; however with the lapse of time, they adapted the routines as part of living there. An informant narrated “Here people live according to a proper plan of meals and sleeping and waking up routine; I did not like it. The environment, way of living, and time plans of meals of the day were different….. but then gradually I got adjusted to the routines”(AA-1).

The participants also expressed their satisfaction as they tried to familiarise in a new atmosphere along with the availability of basic necessities of life such as food, clean water, shelter etc. An informant reflected in these words, “For me this place is as precious as gold. It is God’s blessing for me… If there were no senior citizen homes for old people, we would have to sleep on roads and from where would we have arranged for food” (JM-2). Besides this many of the residents expressed their contentment as they were the victims of loneliness, and were now surrounded by a gathering of people. One of the informants explicated, “There are many people around me in this home. The comfort and peace which I found in this home cannot be compared with anything” (FR-5).

## Discussion

Demographic transitions, coupled with globalization and urbanization, are transforming the economic structure, changing social and cultural values, and weakening of the traditional joint family system. All these factors have had an impact on the lives of the elderly [12, 31]. The changes in the societal and cultural values, distancing in familial relationships, and growing worldly pressures are the significant consequences of globalization [10, 16].

The current study revealed that, in Pakistan the most common reasons for putting the elderly in institutions, was the lack of physical, emotional and financial support especially from their children and their families, migration for better careers, women participating in the workforce, and change in cultural value system. Another considerable reason which this study uncovered and also aligned with a study could be intergenerational inconsistencies between the ageing parents and the adult children resulting in children’s unwillingness to provide all types of support to their older parents [32]. It also discovered unsympathetic behaviour of the children, where the elderly participants were sometimes not even provided with a piece of bread for their sustenance and other basic requirements.

Provision of emotional support is significant to make the elderly feel valued, respected, and cared. The study participants felt mistreated and devalued due to lack of emotional support from their children and family. These findings are in line with the findings of the study by Dubey et al. where 27% of the respondents felt neglected and unimportant, while 3.3% of the informants were demeaned by their daughters-in-law and had to endure the non-caring behaviours of their sons [31]. This negligence generated feelings of isolation, insecurity, low morale, and loss of dignity and self-worth among the elderly. A study done in district Gujrat, Pakistan, found that 80% of the elderly informants were ignored by their family and this was the main cause of loneliness among them [33].

In Asian cultures, women are considered as primary care takers of the family and the elderly, and are also expected to fulfil diverse house hold obligations. After marriage, it is the duty of a daughter-in-law to take care of her parents-in-law [34, 35]. Our study found and confirmed that socio economic pressures and involvement of women in outdoor jobs has not only changed the attitudes towards the female role in Pakistan but has also increased the rejection of this role by women.

The official retirement age in Pakistan is 60 years; however, this figure cannot be considered accurate for a country like Pakistan, where the elderly’s work till their last breath. Conditions like meagre pensions, lack of gratuity and provident funds, and low socioeconomic background forced many of the elderly to search for work [9, 15]. The current study reports that over half of the participants only received education up to the primary level. So, while the elderly were desperate to work and contribute to manage the cost of living and prevent from being financially dependent; unfortunately, they could not do so due to non-availability of jobs and the reluctance of employers in hiring the elderly as their educational level was considered equivalent to that of a manual labour [31, 36].

This study also uncovered certain behaviours depicting resentment, criticism, and intolerance which emerged from frequent family conflicts, intergenerational inconsistencies, poverty, and negativity between parents and children. All this resulted into frustration, loneliness, humiliation, maltreatment, and helplessness among the elderly [37, 38]. Our study outcomes were found compatible with the study done in district Gujrat, Pakistan, where 85% of the participants reported verbal abuse by the children, such as use of harsh words and humiliation [33]. Kim et al. asserts that the stronger the filial bond between the elderly and adult children, the stronger will be the required reaction in terms of exchange of support system [32]. Lack of emotional connection with family, loss of spouse, migration and marriage of children, insufficient resources, and dependency were leading to loneliness, social isolation and psychological distress among the participants which was the important highlights of our study. Loneliness and helplessness were the challenges that surfaced from this study and is corroborated by the study done by Ganatra et al. and Itrat et al.; that these encounters can lead to low self – esteem, insecurity, loss of dignity and depression [9, 39].

There is a strong association between religious beliefs and practices and psychological well-being [31, 40–42]. It strengthens and empowers the individual with courage and positivity. Religious practice was the commonest means of coping used by the informants. A strong religion, faith and regularity in the performance of religious practices assisted the elderly to manage stressors related to lack of money, joblessness, loneliness, rejection, and lack of family support. In addition to prayers, recitation of verses from the holy books, such as The Quran and The Bible, and the reading of religious books were also used.

Though some elders expressed that they felt relaxed and were satisfied, and they got involved in social interaction with other residents, however, while reminiscing about their past life experiences they still missed their children, family, and home, despite their unacceptable behaviours, ignorance and the lack of support from them. Many of them were happy and felt relaxed at getting a proper shelter and the basic needs of life, and they also had a sense of security and freedom from the anxiety and fear of being forced out of the house by their children.

The findings of the study done in Iran well substantiates with our study findings that participants expressed happiness at achieving freedom from regular family issues, rejection, verbal and emotional maltreatment and gained some autonomy [43]. However, others were confronted with undesirable changes in their daily routine and life style, physical location and social interactions and network, which prolonged their adjustment nevertheless, gradually adapted the change. Attachment to their old home and the associated memories were other factors that led them to report feelings of anxiety, helplessness, and social isolation in the care homes.

### Strengths and limitations of the study

Research on this marginalized group is still in infancy in Pakistan. Hence, this study was the first qualitative research done on the older population in Karachi, Pakistan where the elderly were relocated into shelter homes from their own homes by their family and children. The major strength of this study was the descriptive qualitative approach that provided rich insight into the perspectives of older people on the phenomenon of institutionalization. Another was, the maximum variation purposive sampling technique that explored the phenomenon thorough mixed and heterogeneous samples from diverse backgrounds, and contributed genuinely till the saturation was achieved. Also, pilot testing prior to the actual data collection assisted the researcher to interview elderly’s on the sensitive issue. This drill not only empowered her to assess her competency in interviewing but also supported to efficiently tackle the episodes of crying and emotional distress when the participants recalled their tough life experiences.

The study also has some limitations and challenges. The researcher’s lack of understanding of languages other than English and Urdu was the key limitation. Many participants speaking Punjabi, Hindko and Saraiki unveiled their interests to be the part of this study. Their inclusion would have added more richness to the findings. Further, a few of the recognized shelter homes were located in far flung areas of the city, and data collection from these sites would have disclosed other significant themes. However, due to unstable law and order situation in the city and the risk of a female travelling alone to the research sites, the investigator collected the data from the shelter homes which were in close proximity to her residence and university. The researcher faced challenges like prolonged sharing of life stories and reluctance to sit after a certain time due to age related fatigue. As they were fond of sharing their life stories, there were frequent digressions from the topic of discussion, and it was yet another challenge to bring them back on the track of discussion. A few of the informants’ after recalling painful life occurrences, withdrew from the study and were then unwilling to carry on with the interviewing process.

## Conclusion

The results of the current study explicitly supports the emerging phenomenon of institutionalization of the elderly. This concept has emerged from west, where it is considered a usual trend; however, in the eastern countries like Pakistan, this notion is gradually gaining acceptance. To the best of researcher’s knowledge, this study proved to be a significant one, as the true and real experiences of the elderly were investigated who were displaced into the shelter homes by the children and their families. This study also established the criticality of the circumstances these frail elderly people are going through and opened avenues for qualitative and quantitative researches on various issues that were discovered through this study. This study strongly recommends, that there is a dire need for public awareness on care of elderly through different forms of media along with inclusion and implementation of geriatric care in undergraduate and graduate curriculum across all the nursing and medical schools in Pakistan. It is hoped that findings will urge the researchers, HCP, educators, and policy makers to work for the quality of life of ageing group. It is indeed a high time to rejuvenate and revitalize the elderly population and allow them to live their lives with self-respect and dignity.

## Data Availability

The raw data is not publicly available, it can be made available upon request from PI. The recorded data tapes was deleted after transcription, and soft copies were kept password protected. The results of the study did not include any identifiable reference. However, the data obtained from the participants was shared with the research team.

## Abbreviations

CHW: Community Health Workers
ERC: Ethical Review Committee
HCP: Health Care Provider
ICU: Intensive Care Unit
MMSE: Mini Mental State Examination
NGO: Non – Governmental Organization
SPSS: Statistical Package for Social Sciences
SWB: Social Welfare Board
WHO: World Health Organization
